# Supplementary figures and images for: The Role of Extracellular Vesicles in Optic Nerve Injury: Neuroprotection and Mitochondrial Homeostasis
Source: Cells. 2022 Nov 22;11(23):3720. doi: 10.3390/cells11233720 (PMC9738450; doi:10.3390/cells11233720)

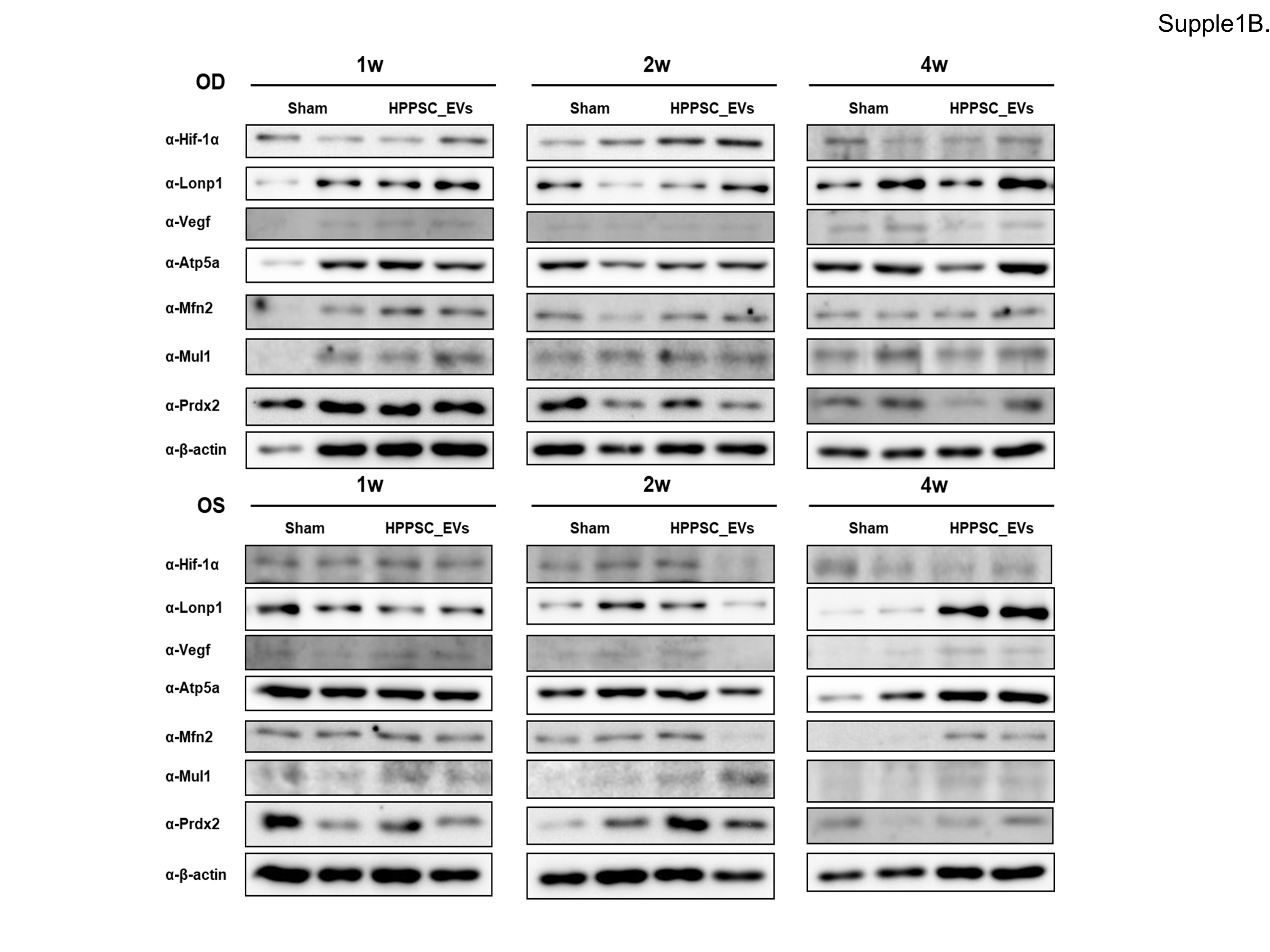

Supplement: Supplementary file 1 [file cells-11-03720-s001.zip › Supple1B.TIF]

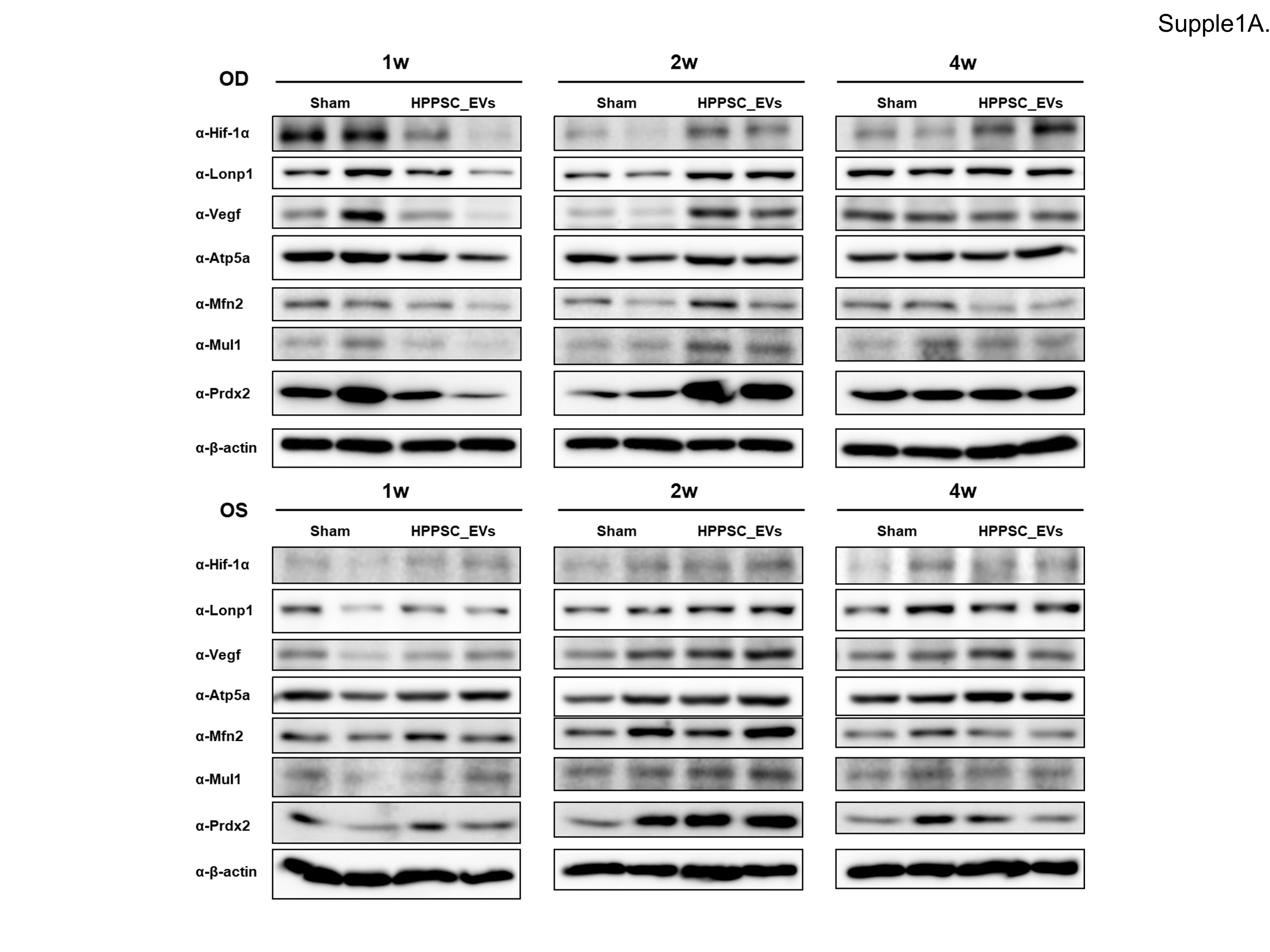

Supplement: Supplementary file 1 [file cells-11-03720-s001.zip › Supple1A.TIF]
